# The Role of the Mizar Mathematical Library for Interactive Proof Development in Mizar

**DOI:** 10.1007/s10817-017-9440-6

**Published:** 2017-11-25

**Authors:** Grzegorz Bancerek, Czesław Byliński, Adam Grabowski, Artur Korniłowicz, Roman Matuszewski, Adam Naumowicz, Karol Pąk

**Affiliations:** 1 Association of Mizar Users, Białystok, Poland; 2 Section of Computer Systems and Teleinformatic Networks, University of Białystok, ul. M. Skłodowskiej-Curie 14, 15-097 Białystok, Poland; 3 Institute of Informatics, University of Białystok, ul. Ciołkowskiego 1M, 15-245 Białystok, Poland; 4 Department of Applied Linguistics, Faculty of Philology, University of Białystok, Plac Uniwersytecki 1, 15-420 Białystok, Poland

**Keywords:** Proof assistant, Repository, Mizar Mathematical Library

## Abstract

The Mizar system is one of the pioneering systems aimed at supporting mathematical proof development on a computer that have laid the groundwork for and eventually have evolved into modern interactive proof assistants. We claim that an important milestone in the development of these systems was the creation of organized libraries accumulating all previously available formalized knowledge in such a way that new works could effectively re-use all previously collected notions. In the case of Mizar, the turning point of its development was the decision to start building the Mizar Mathematical Library as a centrally-managed knowledge base maintained together with the formalization language and the verification system. In this paper we show the process of forming this library, the evolution of its design principles, and also present some data showing its current use with the modern version of the Mizar proof checker, but also as a rich corpus of semantically linked mathematical data in various areas including web-based and natural language proof presentation, maths education, and machine learning based automated theorem proving.

## Introduction

Around 1970s, the advances in computer technology and its popularization together with the proliferation of more user-friendly programming languages allowed the mathematical community to initiate several seminal projects like de Bruijn’s Automath [20], Milner’s LCF [58] or  Glushkov’s Evidence Algorithm [54]. The Mizar project [9] started in 1973 under the leadership of Andrzej Trybulec, first at the Płock Scientific Society and since 1976 at the University of Białystok (formerly the University of Warsaw, Białystok Branch), Poland. From the very beginning Trybulec postulated a language and a computer system for recording mathematical papers in such a way that [35, 56]: (a) the papers could be stored in a computer and later, at least partially, translated into natural languages, (b) the papers would be formal and concise, (c) it would form a basis for construction of an automated information system for mathematics, (d) it would facilitate detection of errors, verification of references, elimination of repeated theorems, etc., (e) it would open a way to machine assisted education of the art of proving theorems, (f) it would enable automated generation of input into typesetting systems. The initial ideas are still valid and with time and a growing support from more researchers involved in the project the current development can be geared towards more ambitious goals, offering more intelligent proof checking methods and better support for the users. A crucial factor that helped to establish Mizar’s position among leading proof assistants, stand the test of time and consequently be developed in the direction of achieving these goals, was the realization of the fact, that large-scale formalizations require developing methods of efficient accumulating, maintaining and re-use of previously generated mathematical content. This approach is today taken by developers of dedicated formal libraries e.g. the Isabelle based Archive of Formal Proofs [14], as well as all large formalization projects, both in mathematics (like the Hales’s Flyspeck project [36], G. Gonthier’s formalization of the Feit–Thompson theorem [25]) as well as in formal computer science (e.g. NASA PVS Library [18], seL4: Formal Verification of an Operating-System Kernel [47], etc.).

## The Beginnings of Mizar Mathematical Library

From the beginning of the Mizar project, the encoding of mathematical proofs was conducted in a dedicated formal language – the Mizar language. The formalization scripts were stored in plain text files, called articles, processed independently and with little connection to one another. The 1981 version of Mizar-2 introduced the environment part of an article, at that time containing statements that were used as axioms and checked only syntactically, i.e., without requiring their justification. Later versions (Mizar-3 and Mizar-4 from the period 1982–1988) divided the processing of Mizar files into multiple passes with file-based communication, such as scanning, parsing, type and natural-deduction analysis, and justification checking. The use of special vocabulary files for symbols together with infix, prefix, postfix notation and their combinations resulted in greater closeness to mathematical texts.

In 1986, Mizar-4 was ported to the IBM PC platform running MS-DOS and later became PC-Mizar in 1988. In the years 1987–1991 the Mizar system and language played an important role in a Polish state research grant programme of the Polish Ministry of Science and Higher Education RPBP III.24 “Logical systems and algorithms for computerized checking of proof correctness”.

It was this project that significantly boosted the development of Mizar articles by numerous authors (about 100 researchers and students from a dozen of scientific institutions from all over Poland), whose efforts resulted in almost 250 Mizar articles. Some of the articles were quite advanced. E.g. Trybulec formalized a 1970 paper by Borsuk *“On the Homotopy Type of Some Decomposition Spaces”* [17]^1^ and his own original result that the algebra of normal forms is a Heyting algebra [83, 84]^2^. Some of the articles resulted from an experimental Mizar-aided topology course where students were supposed to formalize theorems previously introduced informally during lectures. The methodology of this course described by S. Czuba and A. Zalewska in the RPBP III.24 report from 1988 was the following:

Each student had to solve several tasks from a given group of tasks. Initially, it was assumed that the students can re-use in their environment only the theorems and facts previously proved by others. But soon it turned out that this restriction cannot be applied to the simple facts from set theory (very often used in proving topological statements) obvious for students, while at the same time sometimes demanding tedious proofs. Therefore, each task contained in the description of its environment statements of theorems from set theory that were needed to solve a given task.

For each task group a special archive file was created, which was subsequently updated by adding new theorems together with their correct proofs. The care over the file archive was entrusted to one of the students, whose task was to take care of, among other things, the proper order of statements being added, as well as the integrity of operations and relations introduced by the students.^3^

Trying to maintain all these works naturally led to issues concerning the reusability of formalizations. In the RPBP III.24 report from 1988 E. Woronowicz wrote:

In previous versions of the Mizar system, including Mizar-4, the text processed by the Mizar processor consists of two main parts:

- declaration of the environment with definitions of basic concepts (modes, constants, functions) and statements of theorems playing the role of axioms for the developed theory,
- the proper text, which consists of statements of new assertions and their proofs.

Such a text structure, mainly the fact that the user declares the environment, results in his full responsibility for the theory being developed (in the environment there may be contradictory items). Also the preparation time of Mizar articles is lengthened by the fact that when trying to correct errors e.g. in the proof of a statement, the processor processes the correct text of the environment each time. This is not a good solution neither in terms of methodology, nor technology.^4^

The first solution to address the reusability issue and avoid duplication of work was the implementation of a *librarian* utility which helped to include all the notions from previously processed files into a new article without the necessity to copy the whole environment by incorporating the contents of multiple files generated by several passes of the proof checker. This approach, however, turned out to be inefficient for bigger formalizations, which subsequently gave rise to developing methods of exporting only selected items from an article supported by corresponding changes in the Mizar language to facilitate both exporting and importing required notions. Later these concepts evolved into current forms used for importing theorems, definitions and other items from various articles. The functions of the *librarian* utility were superseded by *extractor* and *accommodator*, for extracting semantic information from an article into a database and including this information into a new formalization, respectively. From that time comes the conventional distinction between the library of Mizar articles (as a collection of user-written files in the Mizar language) and the database consisting of the exported semantic information stored in dedicated machine-readable and optimized data formats. It was intended to avoid declaring ad-hoc axiomatics required for proving facts in each article, to help establish “a minimal axiomatization” for a particular article, to reduce the risk of introducing contradictory axioms and repetitions of already proved facts. January 1st, 1989 symbolically marks the date when the current Mizar library—Mizar Mathematical Library (MML) was born as the implementation of that proposal. The (still on-going) process of building one common framework for verifying various branches of mathematics gave rise to a number of subsequent fundamental questions, e.g.:

- should various axiomatizations be allowed or not (and if only one, then which one should be chosen);
- how to build the knowledge database and what information should be stored in it;
- how to organize, manage and ensure the integrity of the database.

In the following sections, we present how these issues have been addressed on the way to developing the current MML.^5^

## Axiomatics

Owing to A. Trybulec’s mathematical background deeply rooted in the Polish school of mathematics, the library was from the beginning based on set theory. However, in the first years of the MML’s existence, there were several experiments with the foundations of the library. Namely, there were attempts to build the library based on set theory with classes (Morse–Kelley) and without them (Zermelo–Frænkel), as well as with the axiom of choice, or without it. Eventually, under the influence of formalizations in category theory, A. Trybulec decided on selecting the Tarski–Grothendieck set theory which is basically the Zermelo–Frænkel set theory with the usual extensionality, pair, union, regularity and replacement axioms, augmented with Tarski’s axiom of existence of arbitrarily large, strongly inaccessible cardinals [80] of the form:

For every set *N* there exists a system *M* of sets which satisfies the following conditions:

(i) $$N\in M$$;
(ii) if $X\in M$ and $Y \subseteq X$, then $Y\in M$;
(iii) if $X\in M$ and *Z* is the system of all subsets of *X*, then $Z \in M$;
(iv) if $X \subseteq M$ and *X* and *M* do not have the same potency, then $X\in M.$

Technically, the initial axiomatization was introduced in two special axiomatic files: HIDDEN and TARSKI. The former contained a selection of primitive notions built into the checker e.g. the types Any and set, equality and membership relations, but also types Element of, Subset of, and the powerset operation bool. The latter presented only the statements of TG axioms. The idea was to keep the proof checking system independent from any particular set theory, and so the type Any was introduced to represent any arbitrary object, not necessarily being a proper set. This, in principle, enabled developing other libraries with different axiomatizations. However, the library developed in terms of TG contained an additional axiom that the type Any is also of type set. Since the interest in developing alternative libraries was rather small, later the type Any was completely removed from the axiomatic files in favor of using the type set alone. Interestingly, in Mizar Ver. 8.1.01 from 2012, to filter out some futile definitional expansions automatically generated by a newly implemented mechanism in the Mizar checker [52], the most general root type was restored into the HIDDEN file, but under a new name: object.

The current form of the TARSKI file representing the set theory axioms is the result of library reorganization performed in connection with exploring fine-grained dependencies in the library [6] that took place in 2013. The original TARSKI file was split into TARSKI_0, TARSKI_A and TARSKI. The TARSKI_0 file contains only ZF axioms, TARSKI_A represents the Tarski’s axiom alone, and the TARSKI file contains more user-friendly formulations of the axioms proved as consequences of the raw axioms from the other two files, e.g. using a defined notion of an ordered set rather than the axiom that such a pair exists.

It should also be noted that the original axiomatics was extended by a selection of properties of some notions commonly used in various formalizations that were built into the system to improve its usability. The extra axiomatic file AXIOMS contained the definitional axioms of the following concepts: element, subset, Cartesian product, domain (non empty set), subdomain (non empty subset of a domain), set domain (domain consisting of sets), as well as the axioms of strong arithmetic of real numbers [76]. The process of restructuring this part of the library had two major steps: first the properties of set-theoretic notions were consequently proved as consequences of basic axioms and moved to ordinary Mizar articles (i.e. ZFMISC_1, SUBSET_1), while the full step-by-step construction of real numbers was consequently defined in the ARYTM* series of articles in the years 1995–1998. The current form of the arithmetic in the MML was shaped around the year 2003 in the course of developing a series of encyclopedic articles XCMPLX* and XREAL* extracted from the library in order to simplify the browsing for selected useful properties of real, complex, and extended real numbers. In consequence, the sets of real and complex numbers were defined together with proving all their usual properties without resolving to any extra axioms, so the original article AXIOMS was removed.

On the other hand, the Mizar system was enhanced by introducing special automation of selected commonly used notions, as so called *requirements* [59]. In particular this concerned the arithmetic of complex numbers, so that the users could decide whether they want the arithmetic facts to be obvious for the Mizar checker [63, 68]. At the same time, the automatically obvious facts found their justification in corresponding ordinary Mizar articles to enable their cross-verification and allow switching off the automation in special contexts, e.g. for educational purposes or for experimenting with axiomatic systems [6].

The separation and consequent use of the minimal axiomatization was an important landmark and since then has been the main approach taken in developing the current MML. This allows to shift the focus in extending the proof checking mechanisms from implementing hard-coded system procedures towards language extensions like *registrations* [62], *properties* [49, 60], or *reductions* [51].

## MML Information Storage

The way in which the information is stored in the MML is a key property of the library. The basic library import/export unit is an article, resembling the conventional publication practice of mathematical papers. Initially, the article’s authors decide which definitions and statements they would like to export and make available to other formalizations, and which should be considered local only. In the process of peer review coordinated by the Library Committee^6^ [30], all exportable items are finally selected and extracted to form the, so called, *abstract*, which contains the bare statements of definitions, theorems, schemes and registrations stripped of all their corresponding proof parts. This public interface information is subsequently stored in the database in dedicated XML-based internal data formats. For any definition, theorem, scheme, or registration, the database contains the corresponding formula represented using *constructors* 7. Different kinds of constructors are used to refer to different classes of defined notions, see Table 1 for the use of constructors and their notations in the current MML.

**Table 1:** The number of constructors and notations

Kind	Number of constructors	Number of notations
Attribute	2890	3237
Functor	8873	9259
Mode	518	1348
Predicate	1137	1304
Selector	187	187
Structure	166	166
Total	13,937	15,667

The information stored in the database which corresponds to a definition, similarly holds a formula (the definiens), but also the definition’s *notation* which consists of its *format* and type information of its arguments, as well as the result type for functor definitions and the mother type for mode definitions. The format of a given definition contains the corresponding symbol (string of characters) together with the information on the arity and position of visible arguments in the text representation. The symbols grouped according to constructor kinds, possibly with additional priority declarations, are stored in, so called, *vocabularies*. Table 2 shows the number of symbols in each category.

**Table 2:** The number of symbols

Symbol	Number
Functor	4825
Attribute	1933
Mode	935
Predicate	746
Selector	175
Structure	168
Left bracket	35
Right bracket	35
Total	8852

The separation of vocabularies from the articles that first introduce given symbols facilitates the use of symbols for multiple, sometimes completely unrelated, notations. Table 3 presents the number of notations for most popular symbols.

**Table 3:** Top 10 symbols wrt the number of notations

Symbol	Notations	Formats	Constructors
.	219	8	219
$*$	197	6	191
$+$	150	6	148
−	141	4	133
Element	65	2	60
@	59	7	59
”	54	3	45
(#)	52	4	51
|	52	3	50
${<}$*	47	8	47

The same format can also be used for arguments with different types, most importantly in the case of the redefinitions of previously defined notions with more specific type restrictions, see Table 4.

**Table 4:** Top 10 symbols wrt the number of formats

Symbol	Notations	Formats	Constructors
$\{$	38	10	38
$\}$	38	10	38
.	233	8	233
${<}$*	49	8	49
*${>}$	49	8	49
@	65	7	65
$*$	207	6	201
$+$	155	6	153
.:	42	6	40
.]	13	6	13

It should also be noted that selected definitions declare special properties of the defined notions to enable further automation in the proof checking software. The information stored in the current database with respect to these properties of predicates, functors and modes is shown in Table 5.

**Table 5:** Properties of predicates, functors and modes

Property	Occurrences	Articles
*Predicates*		
Reflexivity	141	95
Irreflexivity	11	10
Symmetry	125	86
Asymmetry	7	7
Connectedness	4	4
Total	288	202
*Functors*		
Involutiveness	38	32
Projectivity	21	18
Commutativity	161	89
Idempotence	20	13
Total	240	152
*Modes*		
Sethood	8	8

Apart from the theorems, schemes and definitions, the database stores also additional information extracted from the abstracts. That includes presentation related data of new notations (defining synonyms or antonyms for previously defined objects), but also automation, i.e. registrations^8^, as well as term reductions and identifications. Especially the registrations are heavily used in Mizar, see Table 6 (term reductions and identifications are also syntactically represented as registrations).

**Table 6:** The number of registrations

Registration	Number
Conditional	2579
Existential	2871
Functorial	8213
Term identification	152
Term reduction	229
Total	14,044

Since the formalization library imitates to some extent the cumulation of informal mathematical papers, it is interesting to measure the ratio of the number of proved theorems to the number of introduced definitions (cf. [14]). Taking into account only Mizar statements syntactically represented as theorems and schemes, one obtains a ratio of 4.95, see Table 7. However, various forms of Mizar registrations and properties also correspond to theorem statements in informal mathematics. If we also consider them as theorems, the ratio grows to 6.15, see Tables 5 and 6.

**Table 7:** The number of definitions, theorems and schemes

Item	Number
Definition	12,114
Theorem	59,076
Scheme	858
Total	72,048

Importing all the aforementioned information from the database to the environment of new articles is done with the help of a set of designated directives in the Mizar language [34]. A first group of directives enables formulating and disambiguating formal Mizar texts considering overloaded notations i.e. vocabularies, constructors, notations, and partially also registrations and requirements (because of the overloading, the order of used notations is important [66]). Another group of directives concerns encoding proofs, i.e. specifying their skeletons (definitions) and conducting reasoning steps to justify a given goal (theorems and schemes). The rest of import directives influence the way in which the Mizar proof checker uses its built in automation procedures that offer shorter justifications of selected proof steps. This includes the directives requirements, registrations (also importing term identifications and reductions), expansions, and equalities (that automatically provide references to the definitions of used atomic formulas and terms, respectively) [26].

Various independently processed import directives in the Mizar language allow controlling the amount of imported information and accessing only the necessary items from the database. Thanks to this approach, processing information from the database should be similarly time-consuming, despite the level of complexity of the formalization environment.

## Organization and Management

The development of the MML has been driven by several main factors. Initially, most formalization attempts were targeted at covering background knowledge in chosen fields of mathematics. When the library was advanced enough, it was also possible to start works directed at formalizing individual theorems with non-trivial proofs. Moreover, several projects aimed to prove whole papers and monographs were carried out.

The first 3 years of the MML development were dominated by the first approach. The articles developed at that time covered mainly sets, relations, functions, and also geometry and general topology. However, there were also a few articles concerning category theory, functional analysis or quantum theory. Table 8 presents the number of articles (NOA) from that period according to the MSC classification scheme.

**Table 8:** Main fields developed during the first 3 years of the MML

#	MSC	MSC category	NOA
1	03	Mathematical logic and foundations	70
2	51	Geometry	27
3	26	Real functions	23
4	54	General topology	16
5	15	Linear and multilinear algebra; matrix theory	13
6	06	Order, lattices, ordered algebraic structures	11
7	11	Number theory	9
8	14	Algebraic geometry	9
9	18	Category theory, homological algebra	9
10	20	Group theory and generalizations	7
11	12	Field theory and polynomials	6
12	16	Associative rings and algebras	6
13	05	Combinatorics	5
14	40	Sequences, series, summability	4
15	46	Functional analysis	4
16	57	Manifolds and cell complexes	4
17	60	Probability theory and stochastic processes	3
18	28	Measure and integration	2
19	33	Special functions	2
20	13	Commutative algebra	1
21	32	Several complex variables and analytic spaces	1
22	68	Computer science	1
23	81	Quantum theory	1
		Removed	24
		Total	258

It should be noted that the formalizations from these initial years were mostly done independently with no actual connection between formalized theories (e.g. the hierarchies of functions and relations were disjoint, various geometries were constructed in different formal frameworks, groups, rings, fields, and vector spaces were all distinct, without any intrinsic hierarchy). Collecting as many articles as possible was the main priority then. It was the development of the library that eventually allowed to identify common works and emphasize on the quality of the formalizations and the organization of the collected library.^9^

A similar classification of the current MML content shows a certain trend, i.e. topology and real functions are still among the best developed domains (see Table 9). Notably, the field of mathematical applications in computer science, with a significant number of articles in the current MML, was hardly represented (there was just one article formalizing the basic concepts of Petri nets).

**Table 9:** Main fields developed in the current MML

#	MSC	MSC category	NOA
1	03	Mathematical logic and foundations	161
2	06	Order, lattices, ordered algebraic structures	110
3	26	Real functions	101
4	54	General topology	100
5	68	Computer science	97
6	14	Algebraic geometry	84
7	11	Number theory	77
8	46	Functional analysis	70
9	15	Linear and multilinear algebra; matrix theory	62
10	08	General algebraic systems	49
11	57	Manifolds and cell complexes	42
12	05	Combinatorics	39
13	51	Geometry	38
14	18	Category theory, homological algebra	33
15	20	Group theory and generalizations	32
16	28	Measure and integration	31
17	94	Information and communication, circuits	26
18	13	Commutative algebra	16
19	60	Probability theory and stochastic processes	16
20	12	Field theory and polynomials	15
21	16	Associative rings and algebras	15
22	65	Numerical analysis	14
23	33	Special functions	13
24	40	Sequences, series, summability	10
25	30	Functions of a complex variable	9
26	55	Algebraic topology	7
27	52	Convex and discrete geometry	5
28	47	Operator theory	4
29	32	Several complex variables and analytic spaces	3
30	91	Game theory, economics, social and behaviour sciences	3
31	41	Approximation and expansions	2
32	58	Global analysis, analysis on manifolds	2
33	22	Topological groups	1
34	39	Difference and functional equations	1
35	81	Quantum theory	1
36	92	Biology and other natural sciences	1
		Total	1290

The largest area covered in the MML (in terms of the number of articles) are mathematical logic and foundations (MSC #03)—the repository contains the model of the Mizar language itself (the construction of the first-order language, propositional tautologies and satisfiability, Gödel completeness theorem), but also the formalization of various systems of non-classical propositional logic, including linear temporal time or Grzegorczyk’s logics. Alongside with the building blocks of Tarski–Grothendieck set theory (descriptive and combinatorial set theory and large cardinals), other systems based on nonstandard membership relation, such as fuzzy and rough sets [33], are also widely represented. The theory of ordered algebraic structures (MSC #06) follows closely the seminal handbook of G. Grätzer (for lattices and orders) and [23] (for domains). MSC #26 devoted to real functions is one of the most fundamental parts of the MML in terms of knowledge reuse, extending in a straightforward way classical set theory—essentially it covers the standard undergraduate course in mathematical analysis. We can point out that general algebraic systems [31] seem to be underrepresented—summing up the number of files from underlying AMS MSC sections (08, 12, 13, 15, 16, 20), we obtain 186 articles formalizing the classical S. Lang’s course of algebra.

Among the formalizations of particular theorems that stimulated the development of the library we can name an early article formalizing a fundamental theorem of functional analysis—the Hahn–Banach theorem [70] submitted to the MML in 1993. Formalizing the fundamental theorem of algebra [57] completed in 2000 served as an example of a parallel development in several different proof checking systems including HOL Light (2001) and Coq (2002). A collaborative effort to formalize an elementary proof of the Jordan curve theorem (JCT) [50] is notable for developing a vast number of facts concerning the two-dimensional real space and properties of special sequences.

Along with the appearance of “The Hundred Greatest Theorems” list published by Paul and Jack Abad [1] and later expanded by Freek Wiedijk^10^ the library gained another important source from which the theorems to be formalized have been selected by various Mizar authors. At the moment of writing this paper, the Mizar system, with a total of 65 verified theorems from the list, is placed at the third position among all systems involved.

As far as developing formalizations of more comprehensive pieces of mathematics is concerned, the most notable example was the project of translating *A Compendium of Continuous Lattices* (CCL) [23] to the Mizar language. It was considered as an important challenge in the spirit of the QED Manifesto [81]. In the introduction to its second edition, *Continuous Lattices and Domains* [24], the authors wrote:

This is also the place to report on an activity of the Mizar project group located primarily at the University of Bialystok, Poland, the University of Alberta, Edmonton, Canada, and the Shinshu University, Nagano, Japan. It is the aim of the Mizar project to codify mathematical knowledge in a database. The codification means the formalization of concepts and proofs mechanically checked for logical correctness. The Mizar language is a formal language derived from the mathematical vernacular. The principal idea was to design a language that is readable by mathematicians, and simultaneously, is sufficiently rigorous to enable processing and verifying by computer software.

Our monograph A Compendium of Continuous Lattices was chosen by the Mizar group for testing their system. Since 1995, the *Compendium* has been translated piece by piece into the language Mizar. As of August 2002, sixteen authors have worked on this specific project; they have produced fifty-seven Mizar articles.

That project stimulated an extensive development of lattice theory, both in algebraic and topological sense, together with relevant category theory results (compare the position of lattice theory in the list in Tables 8 and 9). On the other hand, a formalization of this size, carried out by an international collaborating group of developers, greatly influenced the development of the Mizar system [13]. The system had to efficiently cope with importing multiple theories, the users had to learn how to routinely work with and share a local database, some mechanisms were developed to deal with various representations of related objects in different formalisms (e.g. lattices as algebraic structures and as ordered sets) [28, 32]. Moreover, these developments created the possibility of formalizing selected papers from current research frontier in that field. For example, the well-developed theory of ordered sets served as a basis for translating a paper on better-quasi-ordering countable series-parallel orders (cf. [82] and [74]).

The initial articles in the MML were produced by Polish researchers and students associated around the Mizar developers. The existence of a growing and centrally maintained library allowed a number of researchers from many research groups to collaborate. This in turn, allowed to build diverse parts of the library corresponding to the area of expertise of many people involved in the project. Table 10 shows that 6 out of 10 most productive MML authors came from countries other than Poland. Overall, the current MML contains contributions developed by authors from 18 countries, see Table 11.

**Table 10:** Top 10 authors

Author	Country	Number of submissions
Yasunari Shidama	Japan	153
Yatsuka Nakamura	Japan	135
Grzegorz Bancerek	Poland	124
Andrzej Trybulec	Poland	123
Artur Korniłowicz	Poland	102
Noboru Endou	Japan	92
Adam Grabowski	Poland	66
Piotr Rudnicki	Canada	60
Xiquan Liang	China	48
Hiroyuki Okazaki	Japan	45

**Table 11:** The number of authors by countries

Country	Number of authors
Poland	109
Japan	62
China	38
Canada	10
Germany	10
Russia	4
USA	4
Austria	3
Italy	2
Netherlands	2
Ukraine	2
Belgium	1
Czech Republic	1
Denmark	1
Finland	1
Israel	1
Myanmar	1
Spain	1
Total	253

**Fig. 1:**
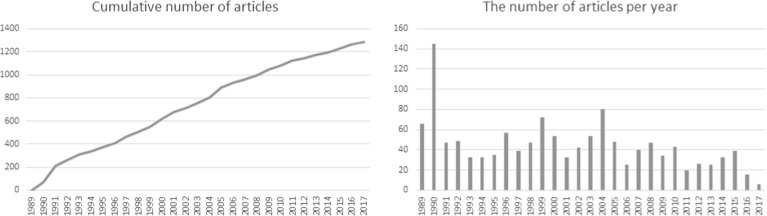
The number of MML articles

The history of submissions presented in Fig. 1 shows an almost linear cumulative number of articles. At the same time one can observe a significantly higher number of formalizations submitted in years 1990, 1999 and 2004. The year 1990 was obviously connected with the intensive work on creating the MML, as well as the fact that a number of formalizations developed with earlier versions of Mizar were converted to the new version used for building the library. In 1999 there were many research groups involved in large-scale collaborative formalizations, including the previously mentioned JCT and CCL Polish–Japanese–Canadian projects. The year 2004 was marked by an intensive development of functional analysis and calculus, mostly by Japanese and Chinese groups.

The interplay of formalizations developed by Mizar users from various groups is seen when analyzing the theorem dependence tree of Mizar articles based on the quantitative information transfer, i.e. the relation induced by the use of the by keyword for straightforward justification within proofs.

In this relation, an article *A* is an ancestor of an article *B* if *B* refers to theorems in *A*, that is *A* transfers information to *B*. The direct ancestor *A* of an article *B* is the article which transfers the largest quantity of information into *B*.

The amount of information that an article *A* transfers to an article *B* is calculated as the sum of information transferred by all theorems from *A* which are referred to in *B*.

Formally speaking, let *T* be a theorem from *A* that is referred to in *B*. The amount of information, *I*, carried into *B* by *T* is calculated using the Shannon formula:

$$\begin{aligned} I = a \left( -\log _2 \frac{n}{N}\right) \end{aligned}$$

where

- *a* is the number of all references to *T* in *B*,
- *n* is the number of all references to *T* in all articles at the time when *B* was the last article accepted into the MML,
- *N* is the number of all references to theorems in the MML at the given time.

In the current MML, the total number of references is 642,978. The article with the maximal number of children (102) is FINSEQ_1 [10] which provides basic properties of finite sequences. The maximal level of the theorem dependence tree is 18, given by the following article chain including formalizations going from the axioms, ordinal numbers, the arithmetic of complex numbers, through Euclidean geometry to planimetry, developed by researchers from Poland, Canada, Japan, China, Italy and Belgium:

18. EUCLID11 “Morley’s Trisector Theorem” by Roland Coghetto
17. EUCLID10 “Some Facts about Trigonometry and Euclidean Geometry” by Roland Coghetto
16. EUCLID_6 “Heron’s Formula and Ptolemy’s Theorem” by Marco Riccardi
15. COMPLEX2 “Inner Products and Angles of Complex Numbers” by Wenpai Chang, Yatsuka Nakamura and Piotr Rudnicki
14. COMPLEX1 “The Complex Numbers” by Czesław Byliński
13. SQUARE_1 “Some Properties of Real Numbers. Operations: min, max, square, and square root” by Andrzej Trybulec and Czesław Byliński
12. XREAL_1 “Real Numbers—Basic Theorems” by Library Committee
11. XCMPLX_1 “Complex Numbers—Basic Theorems” by Library Committee
10. XCMPLX_0 “Complex Numbers—Basic Definitions” by Library Committee
9. ARYTM_0 “Introduction to Arithmetics” by Andrzej Trybulec
8. ARYTM_1 “Non Negative Real Numbers. Part II” by Andrzej Trybulec
7. ARYTM_2 “Non Negative Real Numbers. Part I” by Andrzej Trybulec
6. ARYTM_3 “Arithmetic of Non Negative Rational Numbers” by Grzegorz Bancerek
5. ORDINAL3 “Ordinal Arithmetics” by Grzegorz Bancerek
4. ORDINAL2 “Sequences of Ordinal Numbers. Beginnings of Ordinal Arithmetics” by Grzegorz Bancerek
3. ORDINAL1 “The Ordinal Numbers. Transfinite Induction and Defining by Transfinite Induction” by Grzegorz Bancerek
2. TARSKI “Tarski Grothendieck Set Theory” by Andrzej Trybulec
1. TARSKI_0 “Axioms of Tarski Grothendieck Set Theory” by Andrzej Trybulec

**Fig. 2:**
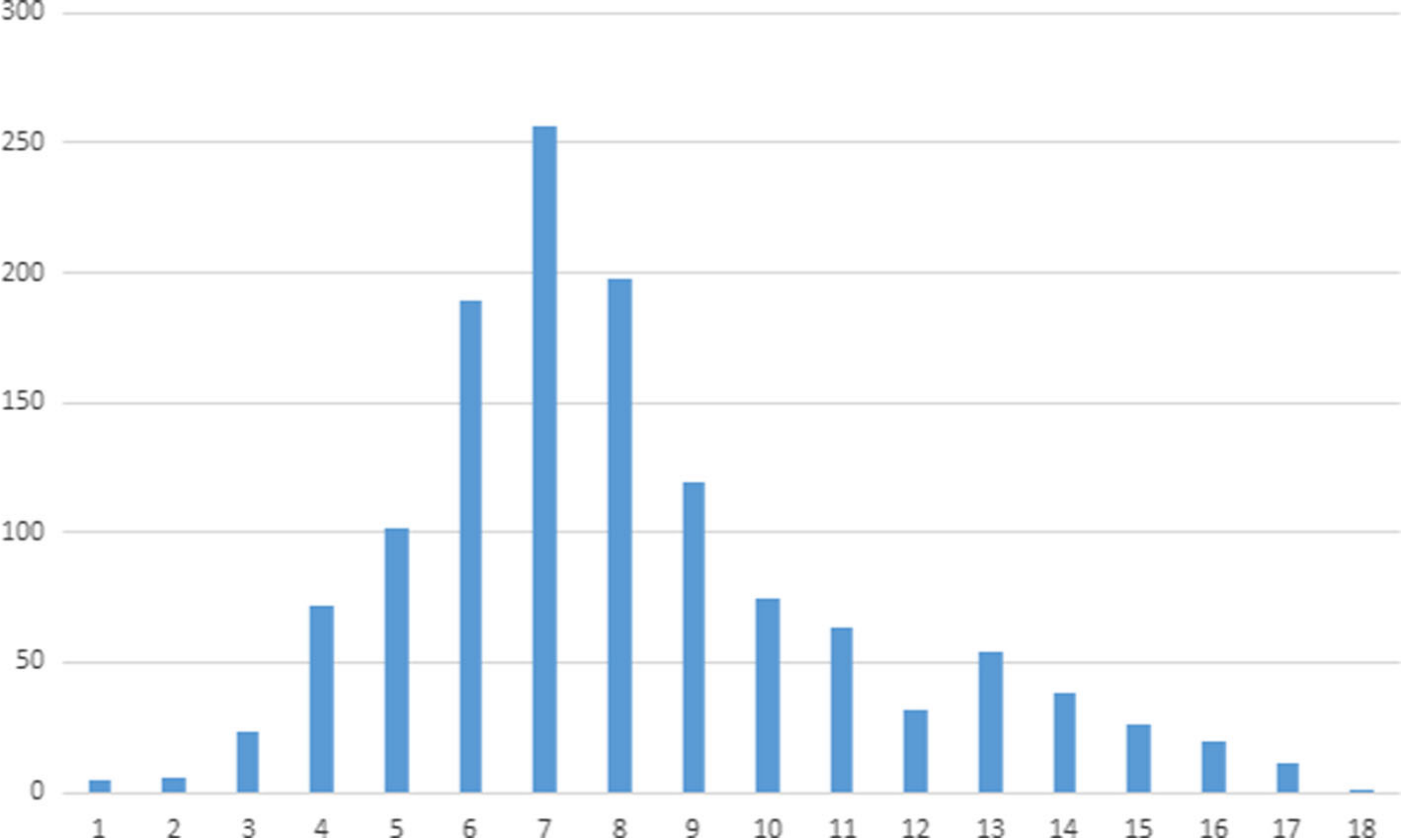
The distribution of the lengths of maximal dependence chains among MML articles

Figure 2 shows the distribution of maximal dependence for all MML articles, i.e. the maximal lengths of chains of dependent articles rooted in a given article.

From its beginnings, the MML was considered an experiment of practical formal modeling of mathematics in order to get a possibly wide group of collaborators. Hence the heterogeneous character of submissions was always taken into account, even if initially most of the authors were from one research group. When the number of collected texts allowed for more statistical methods of information management and the analysis of formalized knowledge, and when the complexity of encoded mathematics went higher and higher, the duties of the Library Committee evolved from a very liberal policy to accept virtually all submissions from the developers “as is”, through the stage of enhancing them in order to get possibly high level of uniformity, into the direction of gradual, continuous, and intensive mechanism of changes of the MML, called *revisions*. One may consider at least four different kinds of revisions:

- an authored revision—consists of small changes in some articles in the library when the author of a new submission notices a possible generalization of already existing theorems or definitions. For such a task, usually it is necessary to improve some older articles that depend on the change. As a rule, however, a rather small part of the library is affected.
- an automatic revision—takes place frequently whenever either a new revision software is developed (e.g. software for checking equivalence of theorems, which enables removing one or two equivalent theorems), or the Mizar verifier is strengthened and existing revision programs can use it to simplify articles [64, 69] or utilize newly implemented language features [53].
- pretty-printing—if changes touch only the parts which are not exported to the library; when newly designed mechanisms allow shorter proofs [71–73].
- a reorganization of the library—it involves changing the order of article processing used for (re-)creating the Mizar database—the ordering is stored in a special file mml.lar distributed with each MML version.

In 2001, a significant reorganization was implemented [78]. Its purpose was to group together all the articles which did not depend on the notion of a structure and place their identifiers at the beginning of the mml.lar list. Two MML parts were respectively named as:

- *concrete*, which does not use the notion of structure (set theory, relations, functions, arithmetic and so on);
- *abstract*, i.e. the article STRUCT_0 and its descendants, all of them directly or indirectly using Mizar structures.

Obviously, these two parts were not meant to be completely independent—the concrete part could be used in the abstract one, but not vice versa.

Apart from that, it turned out to be convenient to also separate a part of the articles in which Random Access Turing Machines were modeled (named “SCM part”). Isolating these articles and placing them at the end of the mml.lar list enabled frequent revisions avoiding the need to constantly update the rest of the library.

**Fig. 3:**
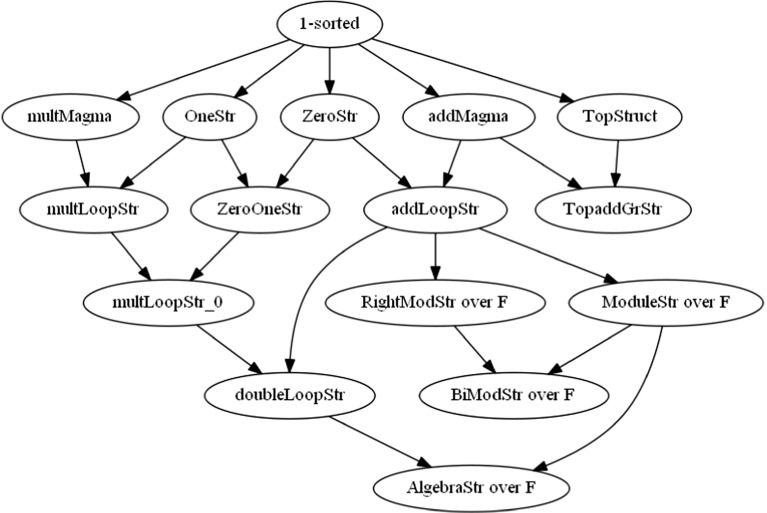
A part of contemporary hierarchy of algebraic structures in the MML

Another part of the library was later distinguished as “Addenda”. Initially it comprised technical articles created in order to prove the correctness of the formal construction of real numbers, previously available as axioms only. This aimed at a gradual reduction of the axiomatic foundations of the Mizar library. Later, this part was extended with auxiliary notions and generalized parts extracted from various articles to enable better reuse of facts within the library. We can mention here the introduction of two big hierarchies of Mizar structures defined retrospectively in a more general way to gain better representation of ordinary mathematical practice [27]: introducing common formal frameworks for both saved 38,649 out of total 447,511 lines of code (LOC), i.e. 8.63% (counting only first 3 years of the MML) and resulted in 24 removed articles (Fig. 3).

A similar approach was taken when the Encyclopedia of Mathematics in Mizar, “EMM”, was formed as another distinctive part of the library in years 2002–2012. Its fourteen articles (with MML identifiers starting with a capital ‘X’) were extracted in order to simplify the browsing for selected, most commonly used notions and their properties (e.g. of real, complex, and extended real numbers, boolean properties of sets, families of subsets, ordered tuples, etc.).

In order to facilitate the process of enhancing the quality of the repository, a reviewing process for submissions to the MML was introduced in 2006. Adopting the commonly used scheme for ordinary mathematical journals accept/revise/reject introduced additional D (for Delay) grade for suggested MML revision. Consequently, the motives for revisions can be for example:

- keeping the repository as small as possible,
- preserving a clear organization of the repository in order to attract authors,
- establishing “elegant” mathematics, e.g. using short definitions (without unnecessary properties) or better proofs.

As one of the the most important MML tools supporting a reviewer’s work, we can point out the MML Query [11] service. It proved its usefulness when subsequent EMM items were created. Also researchers, when writing their Mizar articles, can find it useful, but usually, typical author does not care too much if his lemma is already present in the library. Actually, searching for such auxiliary fact can take much more time than just proving it—this results in many repetitions in the library. This is the area where another tools can be useful: J. Urban’s prototype of a hammer [86] for the MML, called Mizar Proof Advisor, was primarily developed to serve as an assistant for authoring Mizar articles. The fast MoMM (Most of Mizar Matches) tool for fetching matching theorems [87], hence existing duplications can be detected and deleted from the MML [29].

Another popular software, MML CVS—the usual concurrent version system for the MML was active for quite some time, but then was postponed, because the changes were too cryptic for the reader due to the lack of proper marking of items. Actually, one of the most general problems is that there are no absolute names for MML items and the changes are sometimes too massive to find out what really matters; hence the usefulness of the tools of this type is very limited.

Usually, the revision process via automatic generalization of notions improves the MML. There are some problematic issues, however, which suggest that a careful supervision of human reviewers is definitely needed. For example, if we define an even integer number as follows [77]:
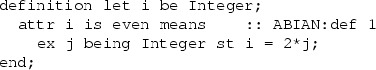


then, quite naturally, we can call *odd* all integers which are not even. As long as the assumption of a variable *i* to be integer is not needed in the formulation of the above definition, it can be marked for removal by the library software (it can be a real number, at least). But then, the number $\pi$ can be shown to be odd as it is not even (although it is not an integer, hence such a classification is void). Any automation of the process of dropping assumption about the types of used loci in the definition of attributes, however useful from a general point of view, could be dangerous.

As a rule, building an extensive encyclopedia of knowledge needs some investment; on the one hand, it can be considered by purely financial means as “information wants to be free, people want to be paid” [2], and it can be observed that projects like the aforementioned formalization of CCL or JCT always go in hand with a significant expansion of the library.

## Other Applications of the MML

The size and the diversity of the MML makes it not only an indispensable standard library for Mizar users, but also an important resource for various projects related to processing mathematical knowledge.

Among the main activities based on the content of the MML, we can mention the development of representations of formal mathematics in human readable LaTeX form (e.g. the *Formalized Mathematics* journal [12]), XML-based semantically-linked web pages [85, 90], more semantic variants of the Mizar language (e.g. WS-Mizar [61]), or general semantically-rich formats like OMDoc [37]. The library’s content available under an open source license [4] was also used as a test bed for developing formal wikis [3]. Moreover, it is worthwhile that the development of first MML-based semantically-linked presentations of mathematical content in the form of the on-line *Journal of Formalized Mathematics* 11 active in years 1995–2004 predated the advent of Wikipedia and other on-line maths journals and services popular today.

As already mentioned in Sect. 2, the development of the Mizar library since its beginnings has been connected with various educational projects involving earlier versions of Mizar. More recently, the organization and scientific content of several courses for mathematics and computer science students which used the modern Mizar versions 7 and 8 have been reported by Retel and Zalewska [75], Borak and Zalewska [16], as well as Naumowicz [65]. Over the last decade, a number of Mizar tutorials at conferences^12^ and at summer schools^13^ have been addressed to students and young researchers. The community of Mizar users has benefited from creating a number of introductory manuals^14^ and a technical reference manual [34]. Some directions in the development of the Mizar proof checking system have also been targeted at beginner users, e.g. the works on making the MML more easily accessible using dedicated auxiliary tools [66] and simplified environment building [67].

Apart from various forms of presenting mathematics, the creation and development of the MML into a large corpus of formalized knowledge enabled experiments with mining the dependencies in formal mathematics [5], as well as training automated theorem provers [88]. The MML served as a basis for preparing large-theory problems to be solved by provers during the competitions of the CADE ATP System Competition [79] and provided a sufficiently big data for machine learning oriented ATP training [21, 40].

Among the most important projects related to the interplay of ATP and the MML we can mention here the J. Urban’s Mizar Proof Advisor, one of the first hammer-style systems i.e. giving the authors of formal proofs a semi-intelligent brute force tool that can take advantage of very large lemma libraries [15]. Thanks to these works Mizar gained ways of cross-verification for its proof checking system by using external provers [89]. This technology was later adopted in the MizAR system which now provides an online automated reasoning service for Mizar users employing a large suite of AI/ATP methods trained over the Mizar library. Reportedly, the system is able to automatically prove 40% of the theorems from the whole MML [45].

The MML is of great value for various machine learning experiments not only for its size, but also for its inherent structure. The application of deep learning techniques [55] taking into account the semantic features of formalized statements significantly improves the premise selection procedure [38] which is at the heart of efficient ATP proof search. The semantic features preserved in the MML, in particular intermediate proof steps, can be used to select interesting lemmas for ATP proofs [42, 44]. Machine learning methods tested over the MML were also applied to define metrics between proofs [8, 41] as well as to align concepts across the libraries of different proof assistants [22]. There were also attempts to translate the MML contents to the formalisms of other proof systems [39].

Finally, it is worthwhile to mention most recent progress in parsing informal mathematics where machine learning methods refer to the correspondence of the formal MML articles and their informal English rendering automatically generated for the *Formalized Mathematics* journal [43].

## Conclusion

To recapitulate the milestone role of establishing and developing formal libraries, the Mizar Mathematical Library in particular, let us recall a remark by F. Wiedijk, which is very accurate in the context of the MML:

We claim that the library is much more important [than] the system. A good system without a library is useless. A good library for a bad system still is very interesting (the system might be improved or the library might be ported to a different, better, system). So the library is what counts. [92]

## Footnotes

1
http://mizar.uwb.edu.pl/milestones/borsuk.pdf

2
http://mizar.uwb.edu.pl/milestones/heyting.pdf

3
http://mizar.uwb.edu.pl/milestones/topology.pdf

4
http://mizar.uwb.edu.pl/milestones/librarian.pdf

5 Current MML Ver. 5.40.1289 is distributed with Mizar system Ver. 8.1.05.

6 The Library Committee of the Association of Mizar Users was officially established on November 11, 1989 with the main aim to collect Mizar articles and to organize them into a central repository (called Main Mizar Library at that time as multiple repositories were supposed to be created, being a direct successor of Central Archive of Mizar Texts coming with Mizar-4).

7 A constructor is a unique number representing a given formal object with respect to its article’s environment.

8 In Mizar, a registration is an umbrella term for three kinds of automation techniques concerning the use of notions represented as adjectives, i.e. existential registrations, conditional registrations, as well as term adjectives registrations, also known as functorial registrations, cf. [34].

9 With time, the initial articles were thoroughly adjusted, 24 articles from that period were entirely removed from today’s library. Overall, the Library Committee decided on removing 52 original articles from the library.

10
http://www.cs.ru.nl/~freek/100/

11
http://mizar.org/JFM

12 E.g. at ISLA 2010 (http://ali.cmi.ac.in/isla2010/workshops/mizar/), CADE 2013 (http://mizar.uwb.edu.pl/CADE23-tutorial/), CICM 2016 (http://mizar.org/cicm_tutorial/).

13 E.g. at the TYPES 2007 Summer School (http://typessummerschool07.cs.unibo.it/#mizar), ESSLLI 2012 (http://www.esslli2012.pl/index.php?id=177).

14 The most recent and up-to-date is F. Wiedijk’s “Writing a Mizar article in nine easy steps” (https://www.cs.ru.nl/F.Wiedijk/mizar/mizman.pdf). Other manuals are available at http://mizar.uwb.edu.pl/project/bibliography.html.
